# Heterogeneous recycled carbon black derived from pyrolytic waste tire rubber with strong, wideband electromagnetic wave absorption

**DOI:** 10.1039/d5ra05326a

**Published:** 2025-09-05

**Authors:** Qirui Sun, Zhongyi Li, Jiaqi Ye, Yuqi Zhai, Xin Ye, Liqun Zhang, Yongpeng Wang

**Affiliations:** a State Key Laboratory of Organic–Inorganic Composites, Beijing University of Chemical Technology Beijing 100029 PR China yexin@buct.edu.cn; b Engineering Research Center of Elastomer Materials on Energy Conservation and Resources, Ministry of Education Beijing 100029 PR China; c State Key Laboratory of Fluorine and Nitrogen Chemicals, School of Chemical Engineering and Technology, Xi’ an Jiaotong University Xi’ an Shanxi 710049 PR China zhanglq@buct.edu.cn; d College of Materials Science and Engineering, Jilin University of Chemical Technology Jilin 132022 PR China wyp4889@163.com

## Abstract

To contribute to the circular and sustainable economy framework, waste tire rubber reclamation by extracting carbon black through pyrolysis and heat treatment and then ingeniously designing it as an electromagnetic wave absorbing (EWA) material is proposed herein. The results showed that the pyrolysis-recycled carbon black (RCB) was heterogeneous with multiple interfaces, making it suitable for EWA application. The RCB was processed at 500 °C–1000 °C to study the changes in the composite and microstructure as well as the EWA properties. The heterointerfaces, defects and components changed with the pyrolytic temperature, which helped to adjust the conductive loss, polarization loss, impedance matching, and electromagnetic wave (EW) transmission path, thereby obtaining different EWA performances. As a result, RCB500 exhibited a minimum reflection loss (RL_min_) of −35.99 dB at a thickness of 2.5 mm with a low filler loading of 40 wt%. Its widest effective absorption bandwidth (EAB) can reach 3.98 GHz at a low thickness of 1.5 mm. For RCB800, the RL_min_ value can reach −34.47 dB, and the EAB_max_ value can reach 4.14 GHz at a low thickness of only 1.5 mm with a filler loading of only 30 wt%. The absorption mechanism was deeply studied. The results indicated that the carbon black pyrolysed from waste tire rubber with strong, wideband electromagnetic wave absorption can be considered a promising candidate for designing high-efficiency carbon-matrix EWA materials.

## Abstract 2

Pyrolytic waste tire rubber-derived RCB was first used as an EWA material owing to its self-contained ash, numerous heterointerfaces and abundant defects.

## Graphical abstract

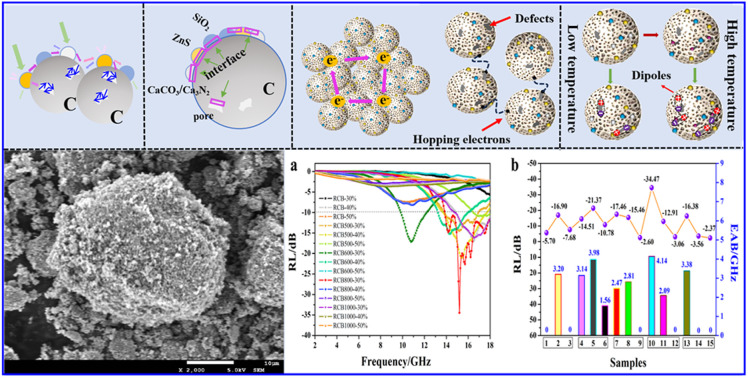

## 1. Introduction

Millions of tires are discarded or buried worldwide annually, posing a very serious threat to ecology. By 2030, 5 billion tires will be discarded regularly.^1^ The traditional methods of landfilling and stockpiling for waste tire management are no longer encouraged from economic, social, environmental, and legal perspectives because waste tires are nonbiodegradable.^2^ Pyrolysis is an efficient and sustainable process that could provide a solution to waste tire disposal problems.^3^ However, one of the major issues behind this practice is the economy of the process and the values of the resulting products. Specifically, the profitability of waste tire pyrolysis at an industrial or semi-industrial scale depends on the market/application of the major products obtained, *i.e.*, the liquid and solid fractions.^4^ The practical use of pyrolysis oil as fuel has been limited due to the high sulphur and aromatic contents and the high cost to upgrade the oil.^5,6^ The solid byproduct of pyrolyzed waste tires, which is called waste tire pyrolysis char, occupies approximately 35–40 wt% of the total weight of waste tires. It consists of carbon black (CB), inorganic compounds (such as silicate minerals, ZnO and ZnS), and trace oil. Among them, CB and ash with heavy residues of pyrolysis oil adsorbed on its surface are the main components in the unprocessed waste tire pyrolysis chars. The unprocessed waste tire pyrolysis chars are mainly used as low-grade reinforcement fillers in the manufacture of low-quality rubber and plastic materials and as pigments for ink.^7^ If the waste tire pyrolysis chars further underwent high-temperature treatment, pyrolysis-recycled carbon black (RCB) would be prepared. The component and the structure would be changed, *i.e.*, production of amorphous carbon, crystalline carbon and ash, pyrolysis of the surface absorbed oil, and formation of a multilayered plane. The RCB is a kind of lightweight carbon substance with high carbon content, high porosity and a large surface area, which is potential to replace commercial carbon black (CB) in certain applications.^8–10^ It is clear that the development of technology not only facilitates the disposal of waste tires with the reduction of quantity, but also focuses on the high-value utilization of their recycled products.^6,11,12^ Thus, we propose an idea to seek an additional method to appraise the intrinsic value of RCB.

Nowadays, with the rapid development of microelectronic technology and instant communication, electromagnetic wave (EW) radiation has become a serious concern for human survival.^13,14^ Long-term exposure to high electromagnetic pollution can lead to many diseases such as dry eyes, joint pain and even intellectual development problems.^15,16^ Therefore, it is essential to design and develop new electromagnetic wave absorption (EWA) materials with high efficiency to protect the human body from the hazards of electromagnetic radiation. Since the 1960s, electromagnetic protection fabrics including metal wire fabrics and metal wire chemical fiber-blended fabrics have been designed.^17^ However, their drawbacks such as high cost, poor flexibility, unsatisfactory electromagnetic protection performance, and processing difficulties limited their development. The desired features of EWA materials include strong absorption over a broad bandwidth, low density, thinness, oxidation resistance, wear resistance, ability to withstand high temperatures and high strength.^18^ However, this has not been completely accomplished so far. Dielectric loss material is one of the main categories in EWA materials.^19^ Among various dielectric loss materials, carbonaceous materials such as carbon nanospheres,^14^ carbon nanotubes,^18,20^ carbon nanofibers,^21,22^ and graphenes^23,24^ are particularly noteworthy for their unique properties of light weight, vast surface area, low density, exceptional dielectric properties, superior conductivity, and remarkable stability.^19,25^ However, the single loss mechanism, impedance mismatch and narrow absorption band resulting from their single component type and simple structures have hindered their practical EWA applications. The conductivity and permittivity of carbonaceous composites can be modified by hybridization with dielectric materials, thus leading to better impedance matching.^26^ As a member of the carbon materials, in addition to the aforementioned advantages, RCB exhibits unique advantages for EWA applications. Unlike other materials requiring hybrid processing, RCB inherently contains mixed ash components that can further facilitate energy dissipation. This intrinsic multi-component system enables efficient EW attenuation without additional hybridization reactions, making RCB a promising candidate for EWA. RCB is a composite that contains ash components such as ZnS and SiO_2_. This has been verified in previous studies. Interestingly, the components were all able to enhance the EWA properties. Although SiO_2_ is an excellent wave-transparent material, it is a desirable candidate for adjusting the EM parameters and improving impedance matching.^27^ Yue *et al.*^28^ developed a network-like structure formed by silicon-coated carbon nanotubes *via* a sol–gel process, which showed a minimum reflection loss (RL_min_) of −54.1 dB at 14 GHz with a low loading rate of 10 wt% and a low absorber thickness of 1.08 mm, covering an effective absorption bandwidth (EAB) of 2.08 GHz. Their results indicated that the impedance mismatch decreased gradually with the increase in SiO_2_ content. Multiple relaxation loss conditions were also generated by the introduction of SiO_2_. Zhicheng Wang *et al.*^29^ used SiO_2_ fillers as a means to mitigate the shrinkage of SiOC ceramics during pyrolysis and to match the impedance matching properties to enhance their EWA capabilities. Finally, the SiOC/SiO_2_ ceramics achieved an RL_min_ value of −28.10 dB with a thickness of 1.90 mm, and the widest EAB was 6.38 GHz at a thickness of 2.30 mm. ZnS is a typical type of transition metal sulphide that has been demonstrated to have good responses to EW, which leads to unique EWA properties. It has been applied in the field of EWA in recent years. Yunfei Yang^30^*et al.* prepared ZnS/NiS/C composites, which exhibited RL_min_ values of −51.45 dB at 4.72 GHz and −56.69 dB at 11.12 GHz. The EAB reached 3.68 GHz at 1.16 mm. The optimal impedance matching and dissipation capability were obtained by adjusting the ratios of NiS and ZnS, in which ZnS played an important role. Jiawei Ding^31^*et al.* fabricated vertical hollow ZnS nanoarrays on a carbon cloth substrate. The composites achieved an RL_min_ value of −52.5 dB and an EAB of 5.1 GHz when the thickness was only 1.9 mm. The excellent EWA performance was due to the unique hollow ZnS nanoarray structure, which enhanced the interface polarization, multiple reflections, and properties of metamaterials with resonant absorption. Therefore, as RCB is a hybrid material that contains ash, it could have an effective EWA capacity. Moreover, the interfaces between carbon and the ash component can generate additional interface polarization and multi-stage scattering, further enhancing the dissipation ability. It is worth to study the varying graphitization degrees, interfaces and components of the RCB with temperature and their effect on EWA capacity. However, this is rarely reported in the literature.

In this work, recycled carbon black obtained from waste tires were used for the first time as an EWA material. The RCB has the characteristic of extremely low cost, which was conducive to large-scale production. It also provided a green way for a reasonable utilization of waste tires. Different from traditional carbon black, the RCB is a hybrid carbon material without any post-treatment. The RCB has an analogous double-layered structure, with ash dots and crystalline carbon attached on the CB sphere. The structural change in ash with temperature is also studied because it has some influences on the EWA properties. Meanwhile, the extensive interfaces were generated by the hybrid structure, which may lead to the occurrence of interface polarization, charge polarization, and associated relaxation losses in the incident wave, thereby exerting an effect on EWA performance further. The relationship between the change in RCB structure or morphology caused by different temperature treatments and EWA performance was the focus of our research. The results indicated that the RCB series had an outstanding EWA ability.

## 2. Experimental

### 2.1 Materials

Pyrolysis-recycled carbon black (RCB) was supplied by Kaiyuan Runfeng Co., Ltd Paraffin wax was obtained from Sinopharm Chemical Reagent Co. Ltd

### 2.2 Preparation of the RCB/paraffin composite material

**Fig. 1:**
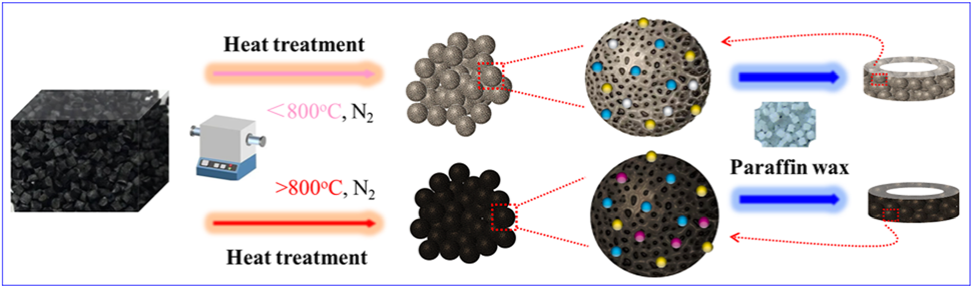
Schematic of the fabrication of RCB composite materials.

The preparation process of the RCB/paraffin composite material is illustrated in Fig. 1. First, pyrolytic RCB was thoroughly ground and placed in a tubular furnace to be calcined at different temperatures in a nitrogen atmosphere for 1 h. The temperatures were set as 500 °C, 600 °C, 800 °C, and 1000 °C, respectively. For convenience, the samples will be mentioned as RCB500, RCB600, RCB800, and RCB1000 hereafter. To prepare thermal cracking carbon black/paraffin composite materials for the research on electromagnetic wave absorption (EWA) performances, paraffin wax was first weighed and poured into an agate mortar, and then thermal cracking carbon black with different grades was added. The mixture was well-mixed through heating, grinding, and ultrasound. It was cooled down and shaped into a regular cylindrical structure with different thicknesses. Black composite bulk with 30 wt%, 40 wt%, and 50 wt% loadings of samples can be obtained. All the above-mentioned operations were very simple and did not require any other chemical treatment. Paraffin wax was used as the substrate because it is transparent to EMW and has no effect on EWA.

### 2.3 Characterization

The morphology and structure of the composite materials were studied using a field emission scanning electron microscope (SEM, JSM-7610F Plus, JEOL, Japan) and a transmission electron microscope (TEM, JEM-F200, JEOL, Japan). The X-ray powder diffraction (XRD) patterns of the prepared composites were recorded using a Siemens X-ray diffractometer (D5005XRD) with Cu Kα radiation (λ = 1.5418 Å) operating at 40 kV and 30 mA to analyze their crystal structure in the scan range of 5°–80° (2*θ*). Thermogravimetric analysis (TGA) was performed using a TGA (TG7, PerkinElmer, United States) from room temperature to 800 °C (which was the upper limit of the instrument) at a heating rate of 10 °C min^−1^ under the flow of N_2_ gas. An FTIR spectrometer (TENSOR, Bruker, Germany) was used to record the Fourier transform infrared spectrum in the range of 400–4000 cm^−1^. The Raman spectra were recorded using a Raman spectrometer (HoribaLabRAM, France) with a 523 nm laser beam as the light source. The specific surface area and porous structure of the materials were analyzed by the Brunauer–Emmett–Teller (BET) method using an automated area and pore size analyzer (JW-BK400). The electrical conductivity was measured using a four-point probe resistivity measurement system (Suzhou Jingge Electronic Co., Ltd, ST2722-SZ, China). To avoid errors, the test was averaged 3 times. The electromagnetic properties of the specimens were analyzed using a network analyzer (VNA, Keysight Technologies, E5071C, USA) through the coaxial method at a band of 2–18 GHz. The samples were cut into a ring shape with an outer diameter of 7.0 mm and an inner diameter of 3.04 mm using a toroidal mold. The reflection loss (RL) was computed using the transmission line theory.

## 3. Results and discussion

### 3.1 Structure analysis of RCB

**Fig. 2:**
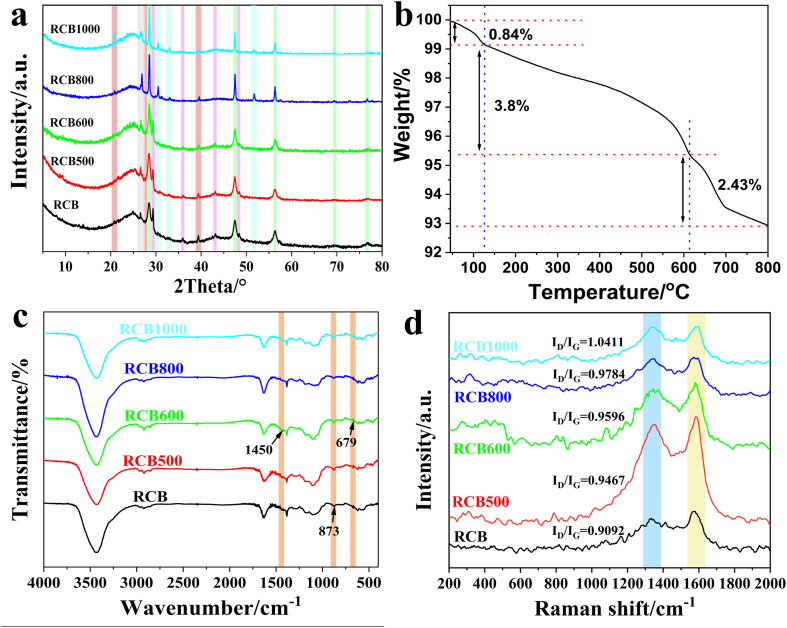
XRD, TGA, FTIR and Raman spectroscopy results: (a) XRD patterns, (b) TGA curve of RCB, (c) FTIR spectra and (d) Raman spectra of RCB, RCB500, RCB600, RCB800, and RCB1000.

The pyrolysis products treated at different temperatures were identified using an X-ray diffractometer (XRD), as shown in Fig. 2a. RCB, RCB500 and RCB600 showed similar diffraction peaks. The presence of amorphous carbon was indicated by a characteristic peak occurring at approximately 25°. The peak observed at 26.6° corresponds to the (011) plane of the quartz phase of SiO_2_, as specified by the database standard PDF 85-0794. (The comparison of the sample with the standard cards for each compound contained in the sample is given in Fig. S1.) The observed reflections at 2*θ* values of 28.5°, 47.5°, 56.4°, 69.4°, and 76.8° matched well with the (018), (0132), (1124), (2032), and (0240) reflections of ZnS, respectively, according to the database standard PDF 89-2201. The reflections at 29.4°, 35.9°, 43.1°, and 48.5° indicate, respectively, the (104), (110), (202), and (116̄) planes of the typical calcite (CaCO_3_; PDF 47-1743). The peak at 39.4° can be attributed to the (205) crystal planes of chaoite carbon (PDF 22-1069). After treatment at a higher temperature, the characteristic patterns of amorphous carbon, SiO_2_, and ZnS persisted. The patterns of chaoite carbon were more obvious. Besides the (205) planes, the (111) and (104) planes of the chaoite carbon appeared at 20.8° and 27.7°, respectively. Moreover, the peaks that belonged to CaCO_3_ all disappeared. Additional peaks emerged at 2*θ* values of 30°, 31°, 32°, and 51°. These peaks corresponded to the (600), (610), (401), and (002) reflections of Ca_3_N_2_ (PDF 21-0153), respectively. The XRD results indicated that elevated temperatures facilitated the crystallization of carbon, which was conducive to the ordering of carbon black. The following reactions may occur among CaCO_3_, C, and N_2_ at elevated temperatures, and may ultimately result in the formation of Ca_3_N_2_:CaCO_3_ = CaO + CO_2_2CaO + C = 2Ca + CO_2_3Ca + N_2_ = Ca_3_N_2_

Due to the changes in composition, the EWA properties of the products may differ.

The thermogravimetric analysis (TGA) curve of RCB from room temperature to 800 °C was obtained to further support the analysis of the XRD pattern. The results are shown in Fig. 2b. There were three stages of weight loss. The first weight loss (0.84%) occurred from room temperature to approximately 125 °C due to the dehydration of the starting material. Previously, the RCB raw material had been treated at 430 °C for a while at the factory to prepare for sale. The results indicated that RCB easily adsorbed water vapour from the air, and this water was held physically in the pores.^32^ The second stage at 125 °C–600 °C was mainly caused by the escape of volatiles from the carbonized substances and the partial rearrangement of amorphous carbon structures. Although the product had been treated at 430 °C in the factory, this was not sufficient to thoroughly eliminate the volatiles because of the very short retention time. In the range of 600 °C to 800 °C, a third weight loss (2.43%) occurred. It has been reported^33^ that obvious decomposition of calcium carbonate in N_2_ occurred above 600 °C and this decomposition intensified at 700 °C. Thus, this weight loss corresponded to the decomposition of CaCO_3_. The results demonstrated that in the products treated below 600 °C, CaCO_3_ was present, whereas above 600 °C, CaCO_3_ had decomposed. This result was in accordance with the XRD analysis.

A Fourier transform infrared (FTIR) spectrometer was employed to further confirm the identities of the products. Fig. 2c demonstrates that the five samples exhibited similar spectra with only a few slight differences. The peak at 1450 cm^−1^, which corresponded to the C–O stretching mode of calcium carbonate,^34^ disappeared when the temperature was above 600 °C. The tiny band at 679 cm^−1^ which corresponded to CO_3_^2−^ (ref. 34) also disappeared. Moreover, the band at approximately 873 cm^−1^, which is due to the bending of C–O,^35^ became weak with the increasing temperature. All of the results confirmed the presence of CaCO_3_ in both the RCB and the low-temperature treatment products. Upon reaching a temperature above 600 °C, CaCO_3_ underwent decomposition. The FTIR data were in good agreement with the XRD and TGA results.

Raman spectroscopy provided a precise method for accurately examining the bonding state and defects of carbon atoms, which had a significant impact on EM parameters. Fig. 2d shows the Raman spectra of the RCB series. Two peaks corresponding to the D band at ∼1350 cm^−1^ and the G band at ∼1590 cm^−1^ can be observed. The former peak originated from the vibration modes of six-membered rings, whose activation resonance process involved peculiar electron–phonon interactions mediated by defects such as point, line, plane defects, and dangling bonds.^36^ The latter peak indicated the sp^2^ hybridized carbon atoms in graphite.^37^ Therefore, the value of *I*_D_/*I*_G_ is always viewed as an indicator of the lattice defect density in carbon materials. As depicted in Fig. 2d, the *I*_D_/*I*_G_ values increased from 0.9092 (for RCB) to 1.0411 (for RCB1000) with the increasing treatment temperature. This indicated an increase in the number of lattice defects in the different samples obtained at different temperatures. The increase in lattice defects was mainly due to the removal of the carbon atoms that were previously bound to oxygen-containing functional groups during a redox reaction process, resulting in the creation of vacancies and structural defects. The abundant defects provided more active sites for the other components in the composites, which can effectively improve their EWA performance.

In conclusion, the XRD patterns, TGA curves, FTIR spectra and Raman spectra confirmed that the treatment temperature caused a modification in the structure of the product. The samples treated at low temperatures were composed of amorphous carbon, SiO_2_, ZnS, and CaCO_3_, of which amorphous carbon and CaCO_3_ were not beneficial to the movement of electrons. The samples treated at higher temperatures consisted of crystallographic carbon, amorphous carbon, SiO_2_, ZnS, and Ca_3_N_2_, with crystallographic carbon and Ca_3_N_2_ contributing to enhanced conductivity.^38^ This may lead to a positive effect on conductive loss. Moreover, there are many interfaces between various compounds that can contribute to the occurrence of polarization loss. Thus, it can be speculated that in addition to the great conductive loss, the obtained products may have great polarization loss.

### 3.2 Morphology analysis of RCB

**Fig. 3:**
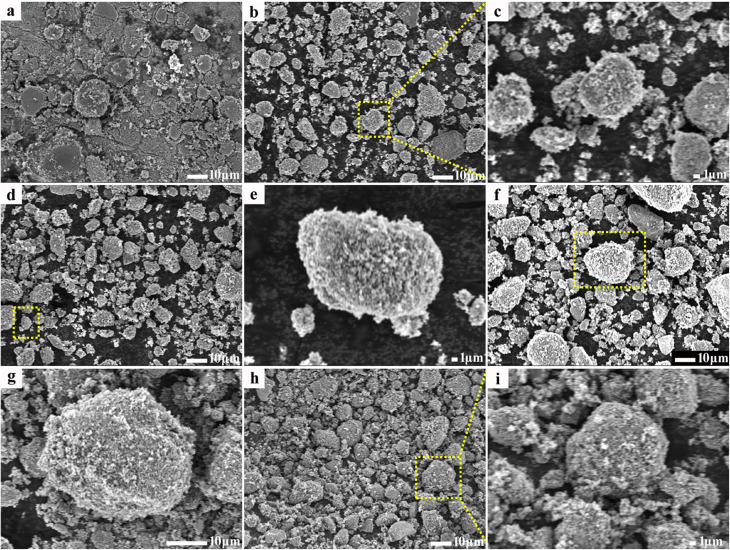
SEM images of different samples: (a) RCB, (b and c) RCB500, (d and e) RCB600, (f and g) RCB800 and (h and i) RCB1000.

**Fig. 4:**
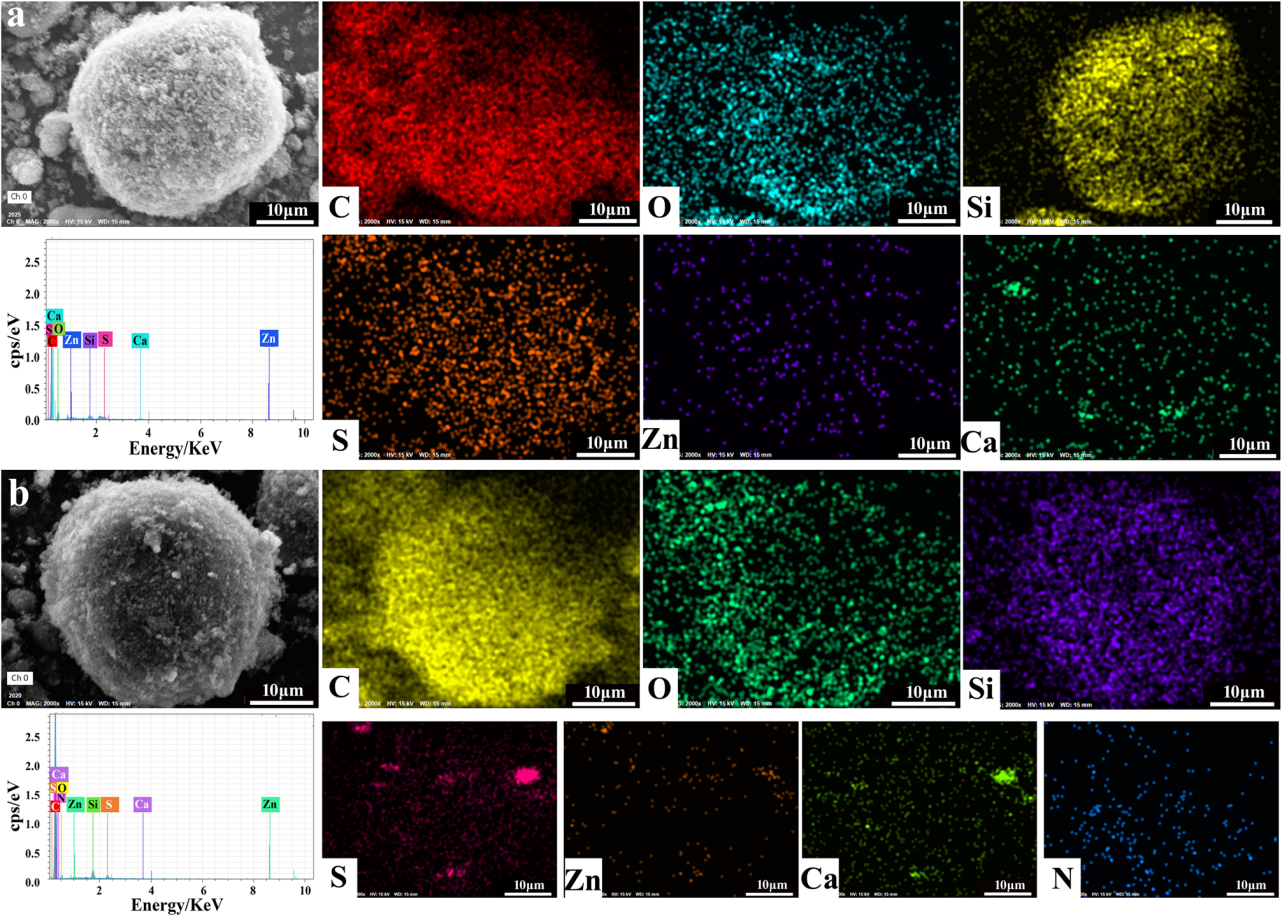
EDS spectra and elemental mapping images of (a) RCB500 and (b) RCB800.

The scanning electron microscopic (SEM) images of the products treated at different temperatures are shown in Fig. 3. All of the samples exhibit a particle morphology, but their surfaces are different. For RCB (shown in Fig. 3a), its surface appears compact and smooth. After treatment at high temperatures (Fig. 3a–i), the surface became coarse and porous. The porosity of the particles increased with the increase in temperature and then decreased. This may be because of the chemical reaction that occurred below 800 °C, which produced some gases that further destroyed the original dense structure. Upon continuously increasing the temperature, in the range of 800 °C–1000 °C, the reaction that produced gas was complete and the structure gradually contracted. Thus, the porosity was decreased. This result agreed well with the BET and TGA results. The nitrogen adsorption–desorption isotherms (Fig. S2) show that with the increase in temperature, RCB develops a microporous structure and reaches a maximum BET surface area of 67.0 m^2^ g^−1^ at 800 °C. When the temperature continues to increase, the BET surface area decreases to 53.8 m^2^ g^−1^. The increase in surface area would increase the multiple reflections and scattering of the incident EW. The elemental mapping images and EDS spectra of RCB500 and RCB800 are shown in Fig. 4. RCB500 and RCB800 are mainly composed of C, O, Si and small quantities of S, Zn and Ca. The only difference between the two samples is that there is N in RCB800. This result is in agreement with the above-mentioned structural analysis. This again proves that CaCO_3_ reacts with C and N_2_ at high temperatures and finally forms Ca_3_N_2_. The magnified images (Fig. 3c, e, g, and i) also indicate that the large particles are composed of numerous tiny particles. To detect differences in the component content of the samples after high-temperature treatment, the atomic percentages of elements are listed in Table 1. Carbon is the dominant element, and its content increases significantly after heat treatment. This enrichment is the primary driver of enhanced EWA performance. The content of all other elements is lower, suggesting that they make auxiliary contributes to the EWA performance. It can also be observed that after high-temperature treatment, the O content decreases, while the Si content increases. In RCB500, O is primarily associated with CaCO_3_ and SiO_2_. The increase in Si content suggests an increase in SiO_2_ content. Thus, the decrease in O content indicates a reduction in CaCO_3_. These results agree with the above analysis. SiO_2_ is an excellent wave-transparent material but can contribute to adjusting the impedance matching. The EWA performance is secondarily influenced by ZnS and Ca_3_N_2_. After treatment at 800 °C, the ZnS content decreases. The decrease may have negative effects on the EWA properties. In contrast, Ca_3_N_2_ is formed from CaCO_3_ at high temperatures and can enhance conductivity. Therefore, based on the changes in elemental atomic percentages, it can be concluded that the improved EWA performance is primarily associated with the dominant C content, the increased SiO_2_ content, and the newly formed Ca_3_N_2_.

**Table 1:** Atomic percentages of the elements in RCB500 and RCB800

Sample	Atom %						
	C	O	Si	S	Zn	Ca	N
RCB500	92.91	3.91	1.83	0.66	0.29	0.40	0
RCB800	94.08	2.84	2.29	0.21	0.11	0.06	0.41

**Fig. 5:**
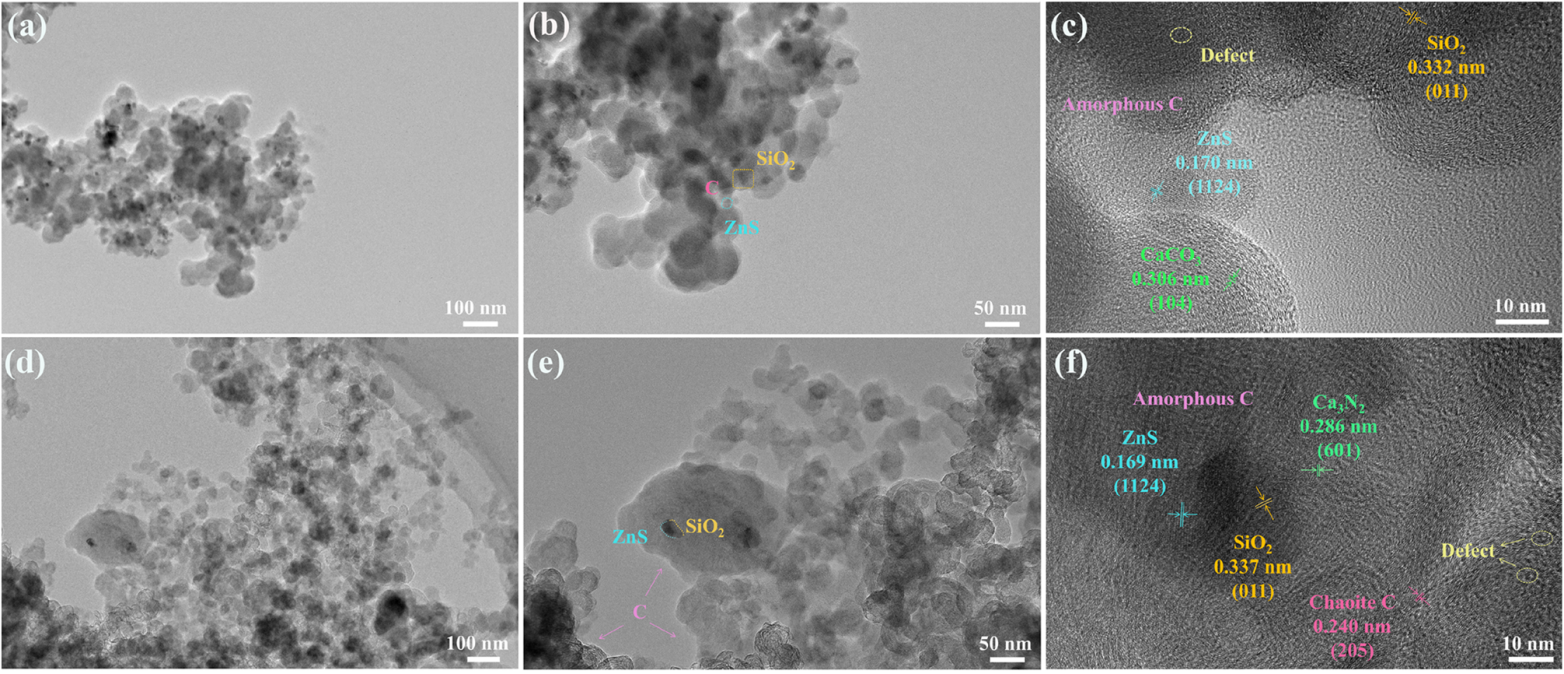
TEM and HRTEM images of (a–c) RCB500 and (d–f) RCB800.

The details of the microstructures were further revealed by transmission electron microscopy (TEM) and high-resolution transmission electron microscopy (HRTEM). The acquired images are shown in Fig. 5 and S3–S8. The large particles are indeed composed of multiple tiny particles, which is in agreement with the SEM results. Fig. S3a and S4 show the morphology of RCB and its corresponding elemental mapping images. Most of the carbon black exhibits a spherical structure with some attached particles. The particles attached to the carbon spheres are composed of Si, O, S, Ca and Zn, as indicated by the elemental mapping images. The elemental mapping results of RCB500 and RCB600 are similar to those of RCB (as illustrated in Fig. S5 and S6), demonstrating that they contain identical components. However, their morphologies are different. With the increase in treatment temperature, the carbon spheres exhibit a progressive increase in their sphericity (Fig. S3a–c), while the boundaries of the spheres become progressively more distinct. There are inevitable spaces between the spheres, which can amplify the multiple reflections and scattering of the incident EW. The HRTEM images of RCB500 are given in Fig. 5a–c. It can be seen that numerous black dots are dispersed throughout the sphere. Upon partial magnification (Fig. 5b), the distinct attachment of dots on the sphere demonstrate the inherent analogous double-layered structure that has many interfaces. The HRTEM analysis results revealed the measured lattice spacings of 0.170, 0.332 and 0.306 nm, corresponding to the ZnS (1124) (JCPDS 89-2201), SiO_2_ (011) (JCPDS 85-0794), CaCO_3_ (104) (JCPDS 47-1743) crystal planes, respectively (Fig. 5c). This demonstrates that the dots are the composition of ZnS, SiO_2_ and CaCO_3_. There are also some areas that lack lattice fringes, which are identified as amorphous carbon. In addition, a lot of defects that can destroy the lattice's periodicity^39^ are presented in the image. They are composed of point defects, lattice dislocations, discontinuous lattice fringes and lattice distortion. These defects can act as polarization centers to assist in creating dipole polarization,^40^ which increases dielectric loss. Meanwhile, the combination of defects, the interface phase, and different substances can form multiple heterogeneous interfaces. This results in enhanced interfacial polarization and multiple EW reflections, which improves the EWA capacity of RCB. After treatment at higher temperatures, the components changed. In addition to the initial elements C, Si, O, S, Ca and Zn, the presence of N was detected (Fig. S7). Fig. 5d–f show the HRTEM images of RCB800, in which the ordered lattice fringes can be observed. The interplanar spacings are 0.169, 0.337, 0.286, and 0.240 nm, respectively, corresponding to the ZnS (1124) (JCPDS 89-2201), SiO_2_ (011) (JCPDS 85-0794), Ca_3_N_2_ (601) (JCPDS 21-0153), and Chaoite C (205) (JCPDS 22-1069) crystal planes. The results verified the XRD analysis that new Ca_3_N_2_ and crystallographic chaoite C are generated at high temperatures. Introducing them enhances dielectric loss through both the material composition and the additional interfaces they create. The areas that lack lattice fringes are caused by amorphous carbon. In addition, defects with a higher density can be observed due to further pyrolysis. The result coincided with the Raman analysis. The elemental mapping images of RCB1000 are shown in Fig. S8, which display similar elemental mapping results as RCB800. The more defects and the more interfaces would further improve the EWA capacity of RCB.

### 3.3 Conductivity

**Fig. 6:**
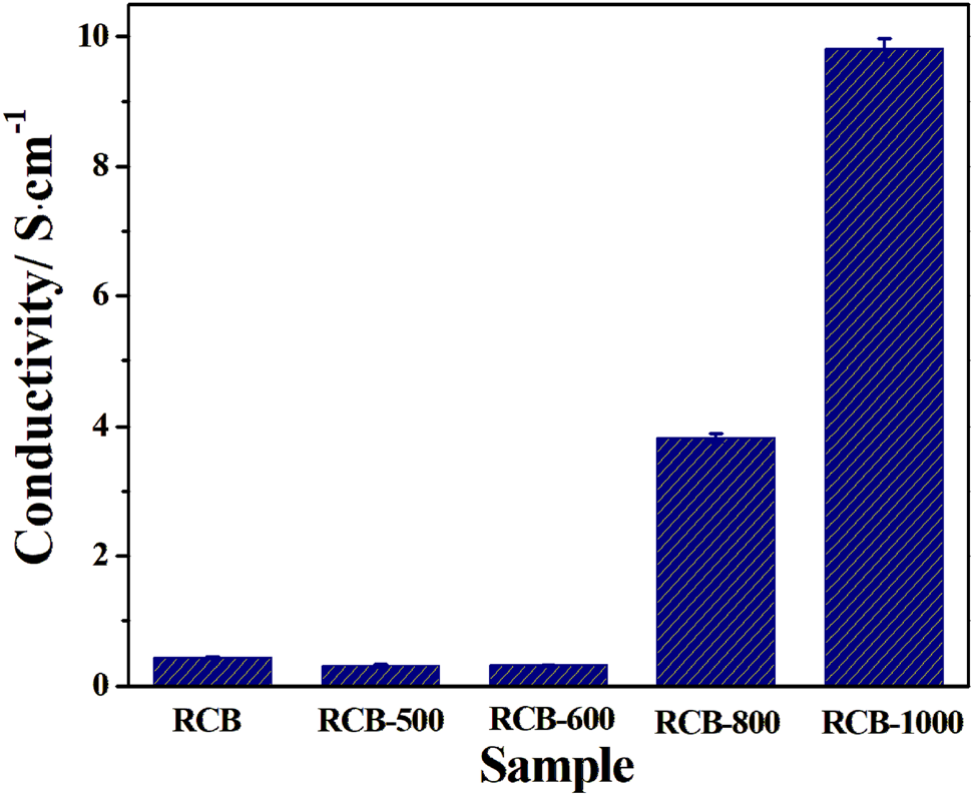
Electrical conductivity of different samples.

The electrical conductivity is a critical factor that defines the conductive loss, which further affects the EWA performance of materials in terms of electromagnetic absorption. As shown in Fig. 6, the conductivity of RCB is only 0.4283 S cm^−1^. Both RCB500 and RCB600 show similar poor conductivities. When the treatment temperature increases to 800 °C and 1000 °C, the conductivity increases. This is due to the changes in the components. Most of the amorphous carbon transforms into chaoite carbon, which has a more orderly crystalline structure and greater conductivity. Meanwhile, the CaCO_3_ solid, which is almost nonconductive, transforms into a nitride with higher conductivity for the considerable porosity absorbing moisture (which has been demonstrated in TGA analysis) to form low-melting surface layers.^38,41^ The results suggest that the high-temperature thermal treatment process can effectively enhance the conductivity, hence improving the conductive loss of the materials.

### 3.4 Electromagnetic (EM) parameters and electromagnetic wave absorption (EWA) properties

First, the EWA performances of all the samples with different loading ratios at a thickness of 1.5 mm were assessed through the reflection loss (RL) parameter, which was calculated from the measured complex relative permittivity and permeability using the following equations based on the generalized transmission line theory and metal backplane model:1
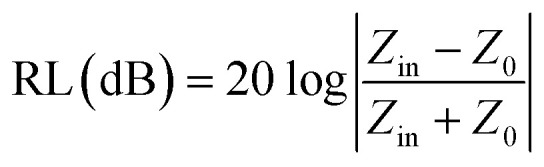

2 *Z*_in_ = *Z*_0_*Z*_r_tan *h*[*j*(2*πfd*/*c*) (*μ*_r_*ε*_r_)^1/2^]

3 *Z*_r_ = *Z*/*Z*_0_ = 1/(*ε*_r_/*μ*_r_)^1/2^

where *Z*_in_, *Z*_0_, *Z*_r_, *Z*, *f*, *d*, and *c* represent the input impedance of the absorbent in the atmosphere, impedance of the free space, impedance matching ratio of the absorbent, impedance value of the absorbent, frequency of the EM wave, coating thickness, and velocity of the EM wave in free space, respectively.

**Fig. 7:**
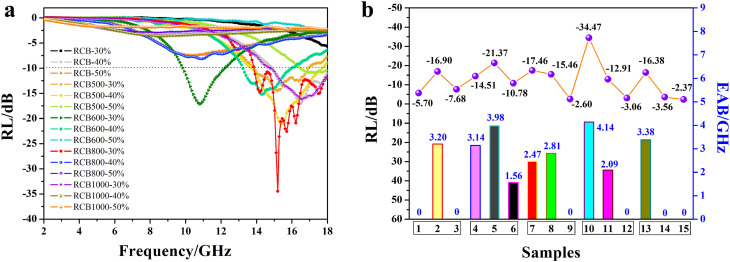
(a) RL-F curves and (b) RL values and EAB values of different samples at a thickness of 1.5 mm. Samples 1–15 in (b) represent RCB-30%, RCB-40%, RCB-50%, RCB500-30%, RCB500-40%, RCB500-50%, RCB600-30%, RCB600-40%, RCB600-50%, RCB800-30%, RCB800-40%, RCB800-50%, RCB1000-30%, RCB1000-40%, and RCB1000-50%, respectively.

The results are shown in Fig. 7. It is obvious that RCB-40%, RCB500-30%, RCB500-40%, RCB500-50%, RCB600-30%, RCB600-40%, RCB800-30%, RCB800-40%, and RCB1000-30% have effective absorption. When the filler loading was only 30 wt%, the absorption intensity initially increased followed by a subsequent reduction as the treatment temperature increased. The RCB800-30% exhibited the highest absorption intensity and the widest EAB. When the filler loading was 40 wt%, RCB500-40% had the best EWA performance from the perspective of intensity and the EAB. Interestingly, all of the samples exhibited a decreasing trend when the filler loading continuously increased, especially for RCB1000. This could be attributed to the increased conductivity, resulting in an impedance mismatch. Therefore, RCB500, RCB800 and RCB1000 were chosen as representative samples for further study to clearly determine the mechanism of EWA performance, especially for the impact of varying treatment temperatures.

**Fig. 8:**
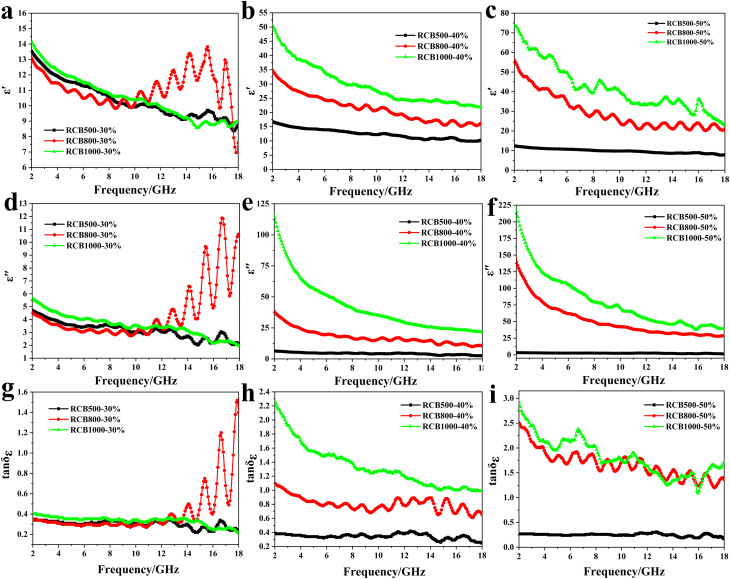
EM parameters of RCB-500, RCB-800 and RCB-1000 with different sample loadings: (a–c) real parts (*ε*′), (d–f) imaginary parts (*ε*′′) of the complex permittivity, and (g–i) dielectric loss tangents (tan *δ*_*ε*_) *versus* frequency.

According to the transmission line theory, EM parameters, which are composed of the complex permittivity (*ε*_r_ = *ε*′ − *jε*′′) and complex permeability (*μ*_r_ = *μ*′ − *jμ*′′), are closely related to EWA properties.^42^ To explore the impact of modifications in morphology and structure on EWA properties and determine the absorption mechanisms, it is necessary to examine variations in EM parameters. Based on the aforementioned structural analysis, it was found that no magnetic compounds were present in the samples. Thus, the magnetic loss could be ignored. Accordingly, it was crucial to thoroughly investigate the real permittivity (*ε*′), imaginary permittivity (*ε*′′), and dielectric loss tangent (tan *δ*_*ε*_). Among the three parameters, *ε*′ represents the capacity of the material to store electrical energy or the degree of response to the EM field, and *ε*′′ represents the ability of the material to lose electrical energy.^43^ The EM values of RCB-500, RCB-800 and RCB-1000 with different sample loadings were measured over a frequency range of 2–18 GHz. Fig. 8 shows the curves of *ε*′, *ε*′′, and tan*δ*_*ε*_ with respect to frequency for different samples.

As shown in Fig. 8a and d, when the filler loading was 30%, RCB800 exhibited the highest average *ε*′ (11.30) and *ε*′′ (4.53) values, followed by RCB1000 (10.80 and 3.70) and RCB500 (10.38 and 3.17). Remarkably, RCB800 had the lowest *ε*′ and *ε*′′ values below 10.1 GHz, whereas beyond 10.1 GHz, the *ε*′ and *ε*′′ values were the highest. This is related to the polarization loss, which is mainly derived from dipole polarization and interfacial polarization in the gigahertz frequency range.^31^ Therefore, dipole polarization occurs at lower frequencies, while interfacial polarization occurs at higher frequencies.^44^ Amorphous carbon tends to provide many defects as polarization centres, which leads to the generation of dipole polarization.^45,46^ XRD analysis reveals that the carbon phase in RCB500 is mainly amorphous, whereas in RCB800 and RCB1000, the carbon phase consists of chaoite and amorphous carbon. The crystallization of carbon is conducive to the ordering of the carbon black atomic structure, which may decrease the number of defects. Therefore, the *ε*′ and *ε*′′ values of RCB500 were initially greater than those of RCB800. However, the atomic arrangement at the interfaces between the crystalline phase and the amorphous phase is disordered. Insufficient atoms exist at the interfaces to form a complete lattice sequence and complete valence bonds, resulting in the presence of suspended bonds. When the temperature increased to 1000 °C, the presence of chaoite phase of carbon became more pronounced, meaning that there were more interfaces between the crystalline and the amorphous phases. Consequently, the number of suspended bonds increased, resulting in a higher number of defects being generated from suspended bonds. Moreover, the TGA curve revealed that the weight ratio consistently decreased as the temperature increased, indicating the occurrence of a redox reaction. The redox reaction that occurred resulted in the removal of a large number of oxygen-containing functional groups, leaving some vacancies at the same position on the carbon. Defect dipoles are generated by the unbalanced charge around the vacancies.^47^ As the temperature increases, the number of defects also increases, which has been proved by Raman and TEM analysis. As a result, RCB1000 exhibited the greatest *ε*′ and *ε*′′ values at lower frequencies. Above 10.1 GHz, interfacial polarization was the predominant factor. The SEM images show that the porosity was the highest when the temperature was 800 °C, which means that most of the interfaces and surfaces were affected, thereby leading to the significant interface polarization. The *ε*′ and *ε*′′ curves of RCB800 fluctuate many times in the high-frequency band, indicating the occurrence of significant polarization loss during the attenuation of incident EW.^48^ The components in the high-temperature-treated products may lead to significant polarization loss. Although SiO_2_ is transparent to EW,^29^ it is a desirable candidate for adjusting the EM parameters.^27^ The heterogeneous interfaces (between SiO_2_ and ZnS and between SiO_2_ and carbon) greatly promote interface polarization. ZnS and carbon have been reported to respond well to EW, which may lead to unique EWA properties. The combination of ZnS and carbon resulted in a closely integrated heterogeneous interface, resulting in powerful interface polarization. In addition, the high-temperature-treated carbon and the produced Ca_3_N_2_ helped to enhance the conductivity, which may have contributed to the loss of conductivity. Notably, as shown in Fig. 8g, RCB800 had a greater average tan *δ*_*ε*_ than that of the other two samples, indicating a greater dielectric loss capability. Although the *ε*′, *ε*′′ and tan *δ*_ε_ values of RCB500, RCB800 and RCB1000 had some differences, the difference was very small.

Significant differences are evident in Fig. 8b, c, e, f, h, and i when the loading ratio was 40 wt% and 50 wt%. It is obvious that all of the values follow to the order of RCB500 < RCB800 < RCB1000. This is attributed to the improvement in the electrical conductivity of the materials with the increase in temperature, as shown in Fig. 6. With the increase in temperature, partial oxygen-containing functional groups were removed from the RCB series spheres due to the redox reaction. The electrically conductive paths for hopping electrons can be reconstructed.^36^ Hopping electrons can jump across defects or between RCB series spheres, which greatly enhances the dielectric loss. In addition, this phenomenon can be attributed to the enhanced interfacial polarization and dipole polarization, both of which can enhance the dielectric loss capability of the samples.^49,50^ The results indicate that a high treatment temperature can effectively enhance the dielectric loss capability of the sample.

**Fig. 9:**
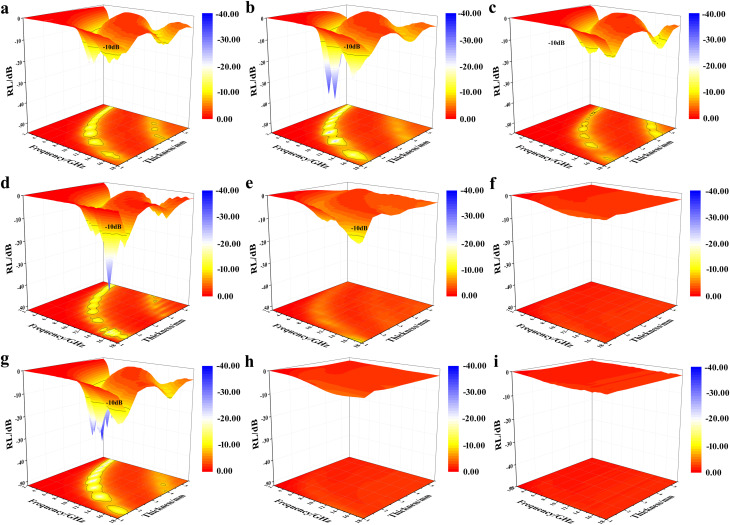
Three-dimensional RL maps in the thickness range of 1.0 mm–5.5 mm for (a) RCB500-30%, (b) RCB500-40%, (c) RCB500-50%, (d) RCB800-30%, (e) RCB800-40%, (f) RCB800-50%, (g) RCB1000-30%, (h) RCB1000-40%, and (i) RCB1000-50%.

**Fig. 10:**
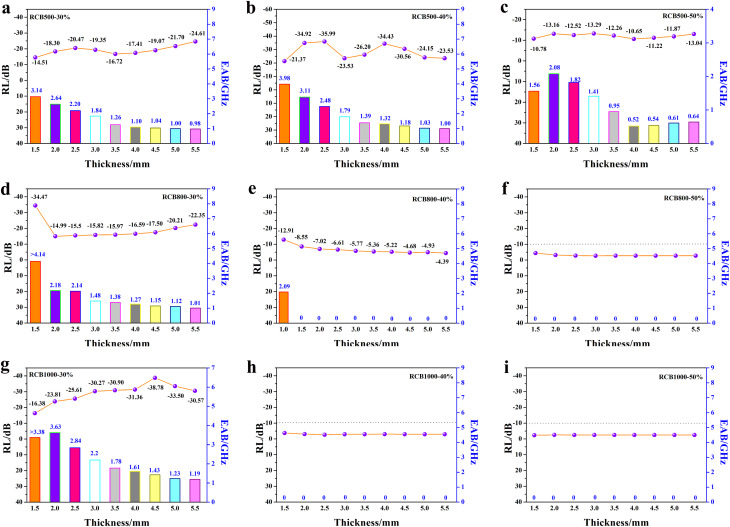
RL and EAB of different samples at different thicknesses: (a) RCB500-30%, (b) RCB500-40%, (c) RCB500-50%, (d) RCB800-30%, (e) RCB800-40%, (f) RCB800-50%, (g) RCB1000-30%, (h) RCB1000-40%, (i) RCB1000-50%.

The reflection loss (RL) values of the composites were calculated using eqn (1)–(3) to evaluate the EWA properties. The results are shown in Fig. 9 and 10. The highlighted parts indicate the regions that are efficient in absorbing. The RCB500 samples, with varying *t* mass ratios and thicknesses in the range of 1.5–5.5 mm, all exhibited effective absorption. Specifically, when the filler loading was 40%, the minimum RL value (RL_min_) reached −35.99 dB with an effective absorption bandwidth (EAB) of 2.48 GHz at a thickness of 2.5 mm. The widest EAB can reach 3.98 GHz at a thickness of 1.5 mm. Unexpectedly, the RCB800 sample showed efficient absorption only at filler loadings of 30% and 40%. Among these, the sample with 30% filler loading exhibited an RL_min_ value of −34.47 dB with an EAB value of 4.14 GHz at a thickness of only 1.5 mm. Effective absorption was observed in the 40% filler loading sample only when the thickness was 1.0 mm, with an RL_min_ value of −12.91 dB and an EAB value of 2.09 GHz. When the filler loading was 50%, no effective absorption was observed. The same trend can be observed for RCB1000. Only when the filler loading was 30%, there was effective absorption. RL_min_ can reach up to −38.78 dB with an EAB value of 1.43 GHz at a thickness of 4.5 mm. The widest EAB value can reach 3.38 GHz at a thickness of 1.5 mm. Nevertheless, with filler loadings of 40% and 50%, there was no discernible absorption. This phenomenon can be attributed to the rapid increase in electrical conductivity with the increase in temperature for the sample. Conversely, because of the higher filler loading, a connected structure was more readily formed to increase the conductivity. Both of these factors can have a negative effect on impedance matching, which may decrease the EWA properties. To meet the requirements of wide, thin and strong absorption performance, it is necessary to minimize the value of RL_min_ and maximize the width of EAB, and the thickness should be as thin as possible. Thus, RCB800 exhibited more pronounced advantages.

It has been reported that two pivotal factors, energy attenuation and impedance matching, should be considered when designing superior absorbing materials.^51^ First, the attenuation factor *α* was studied using the following equation to determine the degree of attenuation:4
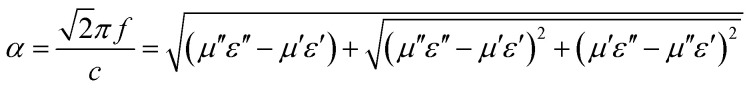


The relationship between *α* and frequency is shown in Fig. S6. It is obvious that the high-temperature-treated products had higher attenuation constant values within the test frequencies. The order was RCB1000-50% > RCB800-50% > RCB1000-40% > RCB800-40% > RCB800-30%. RCB500-30%, RCB500-40%, RCB500-50%, and RCB1000-30% exhibited similar attenuation constant values. The attenuation constant values, which represented the degree of EW attenuation, decreased as the filler loading and treatment temperature decreased. These findings demonstrate that increasing the temperature and loading ratio can effectively improve the attenuation ability. The high attenuation capability was determined relying on the relatively large values of *ε*′ and *ε*′′. However, it is essential to note that a high attenuation constant *α* does not necessarily imply a strong EWA capability.^52^ This is because the EWA capability also depends on impedance matching, as the EM should penetrate the absorber as much as possible.

In order to characterize the impedance matching property of different samples, the value of impedance characteristic parameter (*Z*) used to evaluate the penetration ratio of the EWs and the reflection of EWs at the air–absorber interface was calculated using the following equation:5
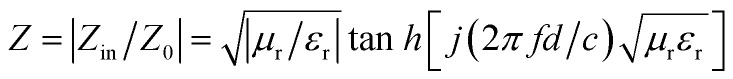
When the value of *Z* is close to 1, which means that the input impedance of the material is close to the free-space impedance, fewer EWs are reflected at the air–absorbing material interface, and more EWs can enter the absorbing material to be lost.

**Fig. 11:**
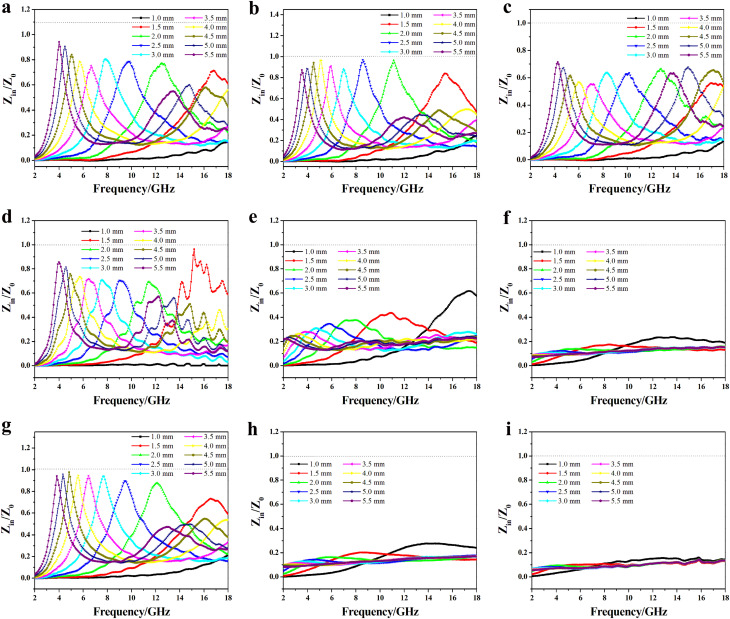
Relationship between *Z*_in_/*Z*_0_ and frequency for (a) RCB500-30%, (b) RCB500-40%, (c) RCB500-50%, (d) RCB800-30%, (e) RCB800-40%, (f) RCB800-50%, (g) RCB1000-30%, (h) RCB1000-40%, and (i) RCB1000-50%.

Fig. 11 illustrates the *Z* values of samples treated at different temperatures and filler loadings. RCB500-40%, RCB800-30%, and RCB1000-30% had *Z* values of 0.9660 at a thickness of 2.5 mm, 0.9639 at a thickness of 1.5 mm, and 0.9777 at a thickness of 4.5 mm, respectively. This demonstrated that the three products had good impedance matching. Therefore, EM waves can penetrate the inside of the material rather than being reflected at the material–air interface. At the other levels of filler loadings, the samples exhibited impedance mismatch, especially for RCB800-50%, RCB1000-40%, and RCB1000-50%. Their values of the impedance parameter exhibited significant deviation from 1, indicating very poor impedance matching, which indicates a higher level of reflection of the EM waves at the interface between the material and air. The impedance matching is related to the characteristics of components and the filler loading. The primary components of RCB500 were amorphous carbon, SiO_2_, ZnS, and CaCO_3_, and CaCO_3_ was not involved in the regulation of the dielectric constant. Therefore, the adjustment of the impedance should depend on the increase in the filler loading. For RCB800 and RCB1000, crystallographic carbon, amorphous carbon, SiO_2_, ZnS, and Ca_3_N_2_ were the main components, and crystallographic carbon and Ca_3_N_2_ can help to adjust the dielectric constant. Thus, only 30 wt% filler loading had good impedance matching. However, when the filler loading was continuously increased, the high conductivity resulted in inadequate impedance matching, causing a higher percentage of EW to be reflected on the surface. Hence, the composites had a high attenuation constant, *α*, but a low EWA capability.

The investigation shows that RCB500-40%, RCB800-30%, and RCB1000-30% exhibited excellent EWA properties, which can be attributed to their good impedance matching. Therefore, the major focus of the investigation was on RCB500-40%, RCB800-30%, and RCB1000-30%.

In order to further examine the EWA mechanism, simulation thickness-F curves were analysed. When the phase difference between the incident and reflected waves in the absorber was 180°, the reflected waves at the air-absorber interface were completely cancelled,^53^ and the reflection of EW on the material surface decreased. This phenomenon is called quarter-wavelength attenuation. This will occur if the thickness of the material meets the following equation at a fixed frequency:6
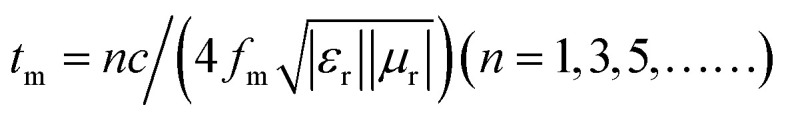


where *f*_m_ and *t*_m_ are the absorption peak frequency and the thickness of the absorber, respectively.

**Fig. 12:**
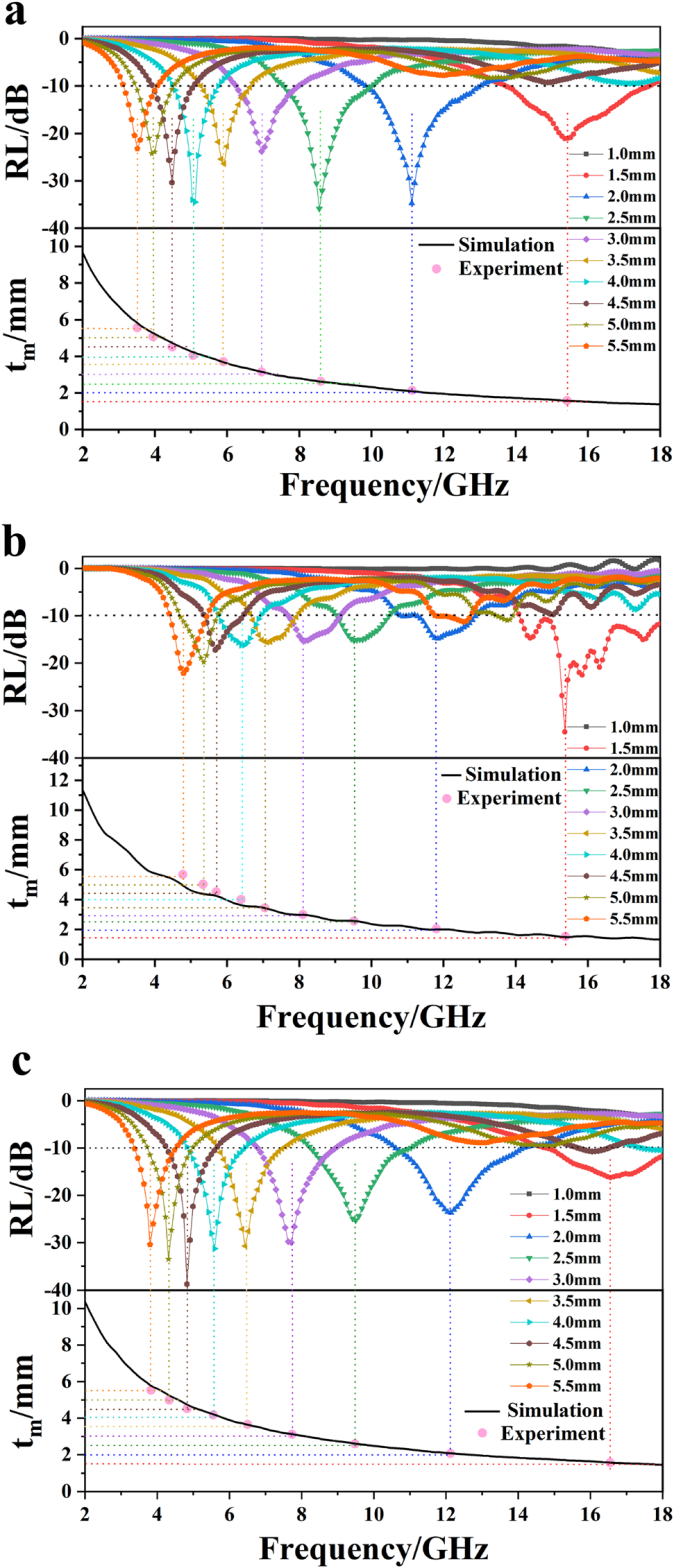
RL-F curves and simulation thickness-F curves of (a) RCB500-40%, (b) RCB800-30%, and (c) RCB1000-30%.

The results are shown in Fig. 12. It can be seen that the experimental values of all of the three samples of *t*_m_ are consistent with the simulated values at 1/4 *λ*, when the thickness was less than or equal to 4.0 mm. However, when the thickness was greater than 4.0 mm, the experimental values deviated from the simulated values. An ideal EW absorbent should be wide, lightweight, thin and strong. Typically, for practical use, the thickness must be less than 4.0 mm. Thus, the deviation that appeared above a thickness of 4.0 mm can be disregarded. The results demonstrate that thin RCB EW absorbers can more easily achieve quarter-wavelength attenuation, hence improving the EWA property. Moreover, it can be concluded from eqn (7) that higher *ε*′ and *ε*′′ values are beneficial for achieving lightweight EW absorbers. The thickness decreased as *f*_m_, *ε*_r_, and *μ*_r_ increased. This indicates that a high treatment temperature would result in a lightweight EW absorber.

In order to understand the efficient EWA of the RCB series, it is necessary to investigate the mechanism of dielectric loss. According to the Debye theory, dielectric losses are mainly composed of polarization loss and conductivity loss, in which polarization loss can be categorized as interface polarization or dipolar polarization.^54,55^ The Cole–Cole semicircle, which was derived from the plot of *ε*′ *versus ε*′′, can be used to evaluate the dielectric loss. Based on the Debye relaxation theory, *ε*′ and *ε*′′ adhere to the following equation:7
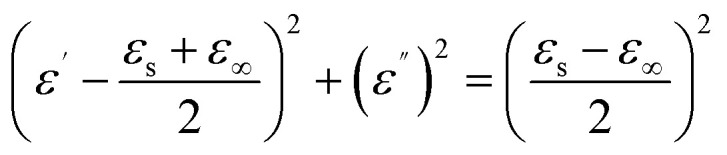


**Fig. 13:**
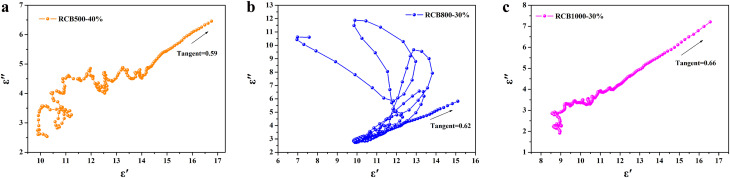
Cole–Cole semicircles of different samples: (a) RCB500-40%, (b) RCB800-30%, (c) RCB1000-30%.

The Cole–Cole semicircles of different samples are shown in Fig. 13. Each semicircle represents a relaxation process, and a larger radius represents a greater relaxation loss.^56^ RCB800-30% exhibited a more pronounced relaxation loss than that of the other two samples. This was mainly caused by the formation of an obviously more porosity structure, which has been discussed in the SEM analysis. In addition, the presence of various heterogeneous interfaces (such as SiO_2_–ZnS, SiO_2_–carbon, SiO_2_–Ca_3_N_2_, ZnS–carbon, ZnS–Ca_3_N_2_, and Ca_3_N_2_–carbon) greatly promoted interface polarization. These substances have different dielectric properties and conductivities. At a heterogeneous interface, accumulated charges caused by the Schottky barrier are formed. Then, they interact with the incident EW to promote dielectric loss.^57^ Moreover, for carbon-based composites, there are many defects and polar groups, which provide conditions for dipole polarization.^58^ Furthermore, the porous structure of carbon networks provides sufficient room for the accumulation of space charges. The decomposition of CaCO_3_ and the formation of Ca_3_N_2_ particles also provided dipole polarization of the dangling bonds around the vacancies. The above-mentioned factors work together to obtain the semicircles. RCB500 and RCB1000 exhibited similar processes. At 500 °C and 1000 °C, there were also many heterogeneous interfaces (such as SiO_2_–ZnS, SiO_2_–carbon, SiO_2_–CaCO_3_, ZnS–carbon, ZnS–CaCO_3_, and CaCO_3_–carbon for RCB500; SiO_2_–ZnS, SiO_2_–carbon, SiO_2_–Ca_3_N_2_, ZnS–carbon, ZnS–Ca_3_N_2_, and Ca_3_N_2_–carbon for RCB1000), as well as dipole polarization provided by carbon. Thus, all of the composites exhibited many semicircles. The multipolarization relaxations caused by heterogeneous interfaces help to enhance the EWA property. However, compared with those of RCB800, the porous structures of RCB500 and RCB1000 were not very obvious, which may have a negative effect on the interfacial polarization, thereby influencing the EWA property. Fig. 13 further demonstrates that there were linear tails in the semicircles of all the samples. The slope of the linear part always represents the degree of conductive loss of the materials. The tangent of the linear part for RCB500, RCB800 and RCB1000 was 0.59, 0.62, and 0.66, respectively, indicating that the high-temperature-treated RCB exhibited great conductive loss. The results confirm the coexistence of dual polarization relaxations and conductive loss.

**Fig. 14:**
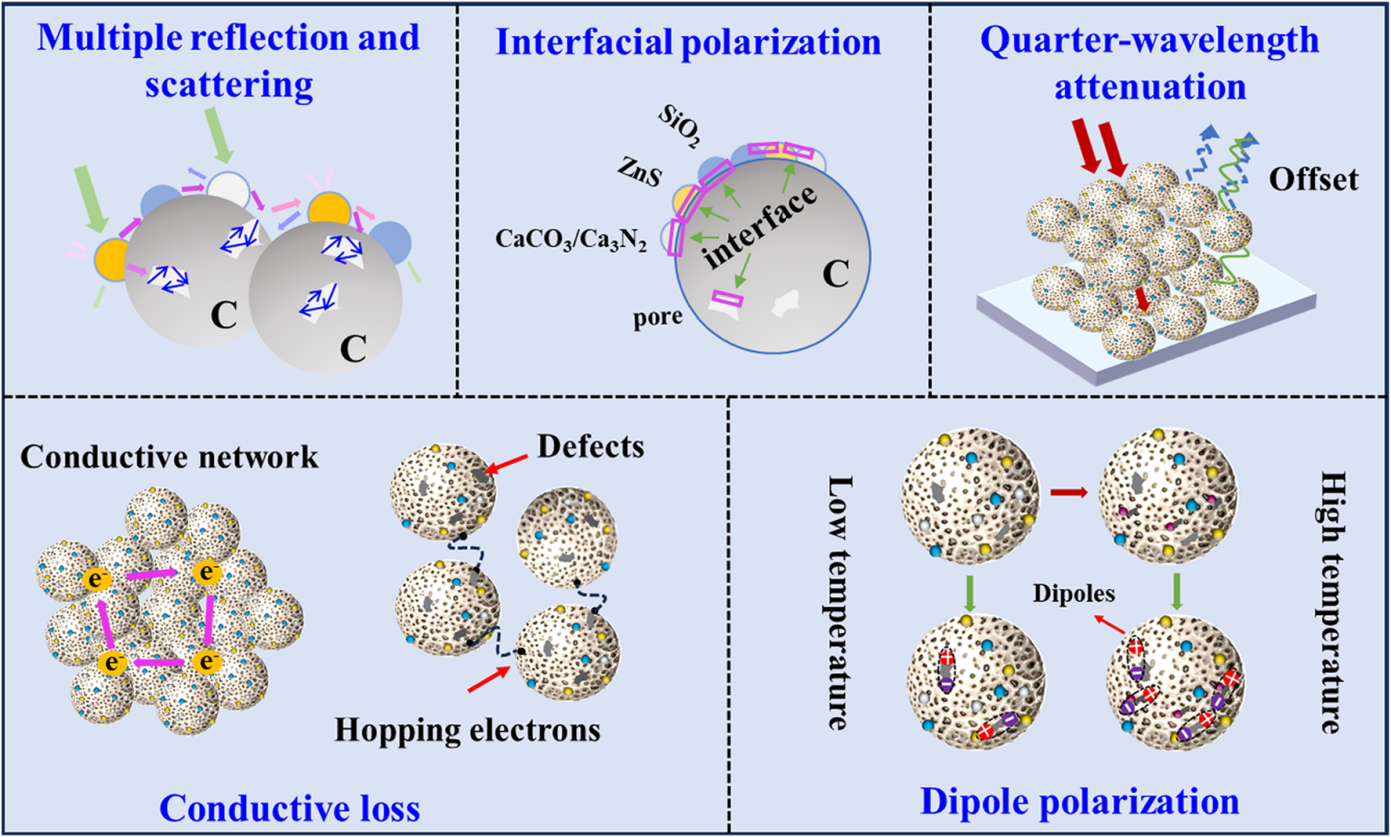
Schematic of the EWA mechanisms of RCB treated at low and high temperatures.

According to the above-mentioned results, the EWA mechanism of the obtained composites can be summarized (Fig. 14). First, the incident EWs experience multiple reflections and scattering inside the porous RCB series significantly extends propagation pathways, thereby intensifying their attenuation.^59^ The morphological characterization confirmed that RCB800 had the greatest porosity. Therefore, RCB800 may exhibit the most pronounced multiple reflection and scattering effects on EWs. Second, the electrical conductivity of the RCB series increased with the increase in treatment temperature due to the phase change of carbon and the formation of Ca_3_N_2_. When an external electric field is applied, the conductivity of the network increases, leading to conductive loss in the composite materials. Moderate high conductivity is usually beneficial to obtain high reflection loss, thus increasing the dielectric loss of the material.^60,61^ Third, apart from the pre-existing defects in the original RCB, new defects were introduced into the composites during subsequent calcination processes at various temperatures. Consequently, dipole polarization is generated as a function of the electric field, in which the positive and negative charge centres of the molecules form an electric dipole moment.^62^ Moreover, SiO_2_ and the newly produced Ca_3_N_2_ are polar molecules^63^ that undergo dipole rearrangement in response to external electromagnetic fields, leading to dipole polarization. Therefore, RCB800 and RCB1000 exhibited an increase in dipole polarization. Fourth, in the RCB series, there were a large number of nonhomogeneous interfaces and heterogeneous junctions, including carbon sphere inner and outer interfaces, pore interfaces, air-different substance interfaces, and SiO_2_–ZnS, SiO_2_–carbon, SiO_2_–CaCO_3_, ZnS–carbon, ZnS–CaCO_3_, and CaCO_3_–carbon interfaces for RCB500 and SiO_2_–ZnS, SiO_2_–carbon, SiO_2_–Ca_3_N_2_, ZnS–carbon, ZnS–Ca_3_N_2_, and Ca_3_N_2_–carbon interfaces for RCB800 and RCB1000. Interfacial polarization is facilitated by these interfaces.^64^ Fifth, the good impedance matching optimized by the components and the filler loading caused the EWs to dramatically enter the interior of the RCB series rather than reflecting at the air–absorbing material interface. The *Z* values confirmed that the impedance matchings of RCB500-40%, RCB800-30%, and RCB1000-30% were better than those of the other samples. Ultimately, the EM reflection was further significantly decreased through quarter-wavelength attenuation, particularly for RCB500-40%, RCB800-30%, and RCB1000-30% when the thickness was less than 4.0 mm.

From the perspective of practical production applications, RCB800 can serve as a promising EWA material. This is because compared to RCB500 and RCB1000, RCB800 achieved the widest EAB (4.14 GHz), strongest loss capability (−34.47 dB), smallest thickness (1.5 mm), and lowest filler loading (30 wt%) at a moderate temperature. The EWA properties of typical carbon-based composites reported in the recent literature are shown in Table 2. The RCB800, developed in this study, had superior EWA capabilities compared to conventional C-based materials, as demonstrated by its higher RL, thinner thickness, wider EAB, and lower filler loading values. The results once again indicate that RCB with further heat treatment can serve as a highly efficient EWA material.

**Table 2:** Reported EWA characteristics of different carbon-based composites

Samples	RL_min_ (dB)	Thickness (mm)	EAB (GHz)	Filler loading (%)	Refs.
Carbon nanofibers	<–25.0	4.0	<2.0	100	65
Fe_3_O_4_@C nanofibers/bismaleimide composites	−22	6.0	≪1	100	66
CuS-biomass carbon	−28.8	4.08	4.2	20	67
Mn–Zn ferrite/carbon fibre	−28.75	10	1.46	100	68
Thermosetting polyimide-derived carbon	−36.5	1.8	4.1	30	69
Reduced graphene oxide	−37.2	3.5	<2.0	30	36
Hollow ZnFe_2_O_4_/residual carbon	−38.99	4.0	3.2	45	70
Carbon-wrapped Y_2_Si_2_O_7_	−42	3.3	3.6	100	71
NiS/Ni_0.96_S@multiwalled carbon nanotube	−35.5	2.0	<3.76	30	72
N, S Co-doped porous carbon	−15.478	2.0	2.08	70	73
RCB800	−34.47	1.5	4.14	30	This work

## 4. Conclusion

In this work, a series of carbonized pyrolytic carbon black samples have been prepared by controlling the pyrolysis temperature. The resulting pyrolyzed materials exhibited different morphologies, different components, and varying degrees of graphitization when the temperature was adjusted. Morphology characterization revealed that the RCB series were porous and composed of many tiny spheres. The porosity increased with the increase in temperature until 800 °C. XRD, FTIR spectroscopy, and TGA analyses revealed that when the pyrolysis temperature was low, the material was composed of amorphous carbon, SiO_2_, ZnS, and CaCO_3_, whereas when the pyrolysis temperature was high, the material was made up of crystallographic carbon, amorphous carbon, SiO_2_, ZnS, and Ca_3_N_2_. Raman spectroscopy analyses indicated that the degree of graphitization of RCB treated at high temperature was greater than that of RCB treated at low temperatures, which was due to structural differences. Moreover, the electrical conductivity increased with the increase in treatment temperature. Reflection loss (RL) measurements revealed that the prepared samples had good electromagnetic wave absorption (EWA) properties at different pyrolysis temperatures. RCB500 exhibited an RL_min_ value of −35.99 dB at a thickness of 2.5 mm with a filler loading of 40 wt%. Its widest EAB was 3.98 GHz at a thin thickness of 1.5 mm. For RCB800, the RL_min_ value was as high as −34.47 dB, and the EAB_max_ value can reach 4.14 GHz at a low thickness of only 1.5 mm with a filler loading of only 30 wt%. With respect to multiple components, including variations in components with temperature, the original abundant carbon provided attenuation ability involving dipole polarization, interfacial polarization and conductive loss. The porous particle-stacked morphology provided a large surface area, heterogeneous interfaces and surfaces, which also had a positive effect on interfacial polarization loss and conductive loss, as well as widening the transmission route of EW. Most importantly, the optimization of the components and the filler loading improved the impedance of the materials. As a result, the EWA properties of the as-prepared samples were superior to those of most recently reported carbon-based composites. This work, on the one hand, lays the foundation for detecting changes in components during heat treatment of pyrolytic carbon black and, on the other hand, provides inspiration for studying the promising EWA application of carbon black obtained through pyrolysis of waste tires.

## Author contributions

Qirui Sun: writing–original draft, writing–review and editing, data curation, visualization, investigation, software, and conceptualization. Zhongyi Li: investigation, methodology, visualization, and validation. Jiaqi Ye: investigation and visualization. Yuqi Zhai: writing–review and editing. Xin Ye: writing–review and editing, funding acquisition, project administration, conceptualization, and supervision. Liqun Zhang: funding acquisition, project administration, resources, and supervision. Yongpeng Wang: writing–review and editing, project administration, conceptualization, and supervision.

## Conflicts of interest

The authors declare that they have no known competing financial interests or personal relationships that could have appeared to influence the work reported in this paper.

## Supplementary Material

RA-015-D5RA05326A-s001

## Data Availability

The data for this article, including all curves, are available at Science DB at [URL-format https://doi.org/10.57760/sciencedb.27951]. Supplementary information: the comparison of the sample with the XRD standard cards for each compound contained in the sample; nitrogen adsorption–desorption isotherms and pore size distributions of all sample; TEM images of RCB, RCB600, RCB1000; TEM elemental mapping images of RCB, RCB500, RCB600, RCB800, RCB1000; attenuation constants of all samples. See DOI: https://doi.org/10.1039/d5ra05326a.
