# Supplementary material for: Heterogeneous recycled carbon black derived from pyrolytic waste tire rubber with strong, wideband electromagnetic wave absorption
Source: RSC Adv. 2025 Sep 5;15(38):31865–83. doi: 10.1039/d5ra05326a (PMC12412126; doi:10.1039/d5ra05326a)
Supplement: RA-015-D5RA05326A-s001 [file RA-015-D5RA05326A-s001.pdf]

Supplementary Information (SI) for RSC Advances.
This journal is © The Royal Society of Chemistry 2025

Heterogeneous Recycled Carbon Black from Pyrolytic Waste Tire Rubber with Strong, Wideband Electromagnetic Wave Absorption

Supporting Information

Qirui Sun [a, b], Zhongyi Li [a, b], Jiaqi Ye [a, b], Yuqi Zhai [d], Xin Ye [a, b *], Liqun Zhang [a, b, c *], Yongpeng Wang [d *]

a. State Key Laboratory of Organic-Inorganic Composites, Beijing University of Chemical Technology, Beijing, 100029, PR China

b. Engineering Research Center of Elastomer Materials on Energy Conservation and Resources, Ministry of Education, Beijing, 100029, PR China

c. State Key Laboratory of Fluorine and Nitrogen Chemicals, School of Chemical Engineering and Technology, Xi' an Jiaotong University, Xi' an, Shanxi, 710049, PR China.

d. College of Materials Science and Engineering, Jilin Institute of Chemical Technology, Jilin, 132022, PR China

* Corresponding author e-mail: yexin@buct.edu.cn (X. Ye), zhanglq@buct.edu.cn (L. Zhang), wyp4889@163.com (Y. Wang)

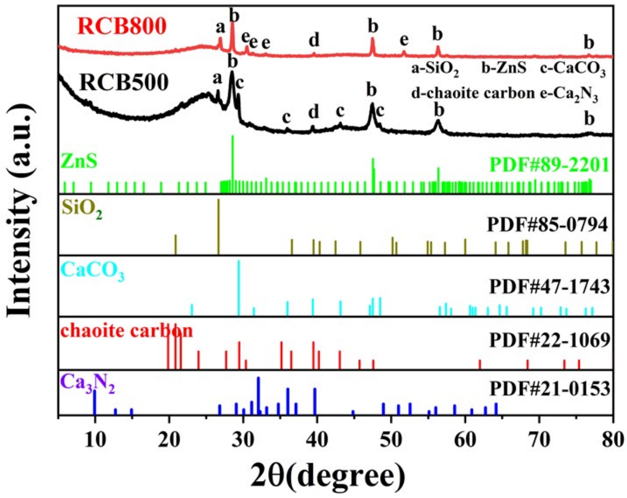

Fig. S1. The comparison of the sample with the XRD standard cards for each compound contained in the sample.

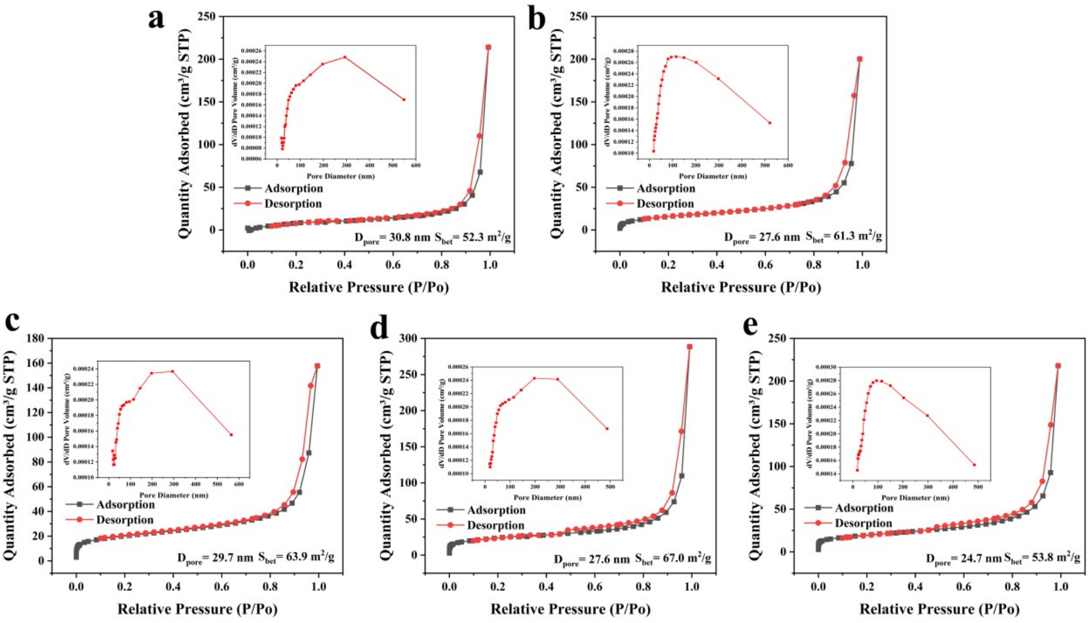

Fig. S2. Nitrogen adsorption–desorption isotherms and pore size distributions of (a) RCB, (b) RCB500, (c) RCB600, (d) RCB800, (e) RCB1000.

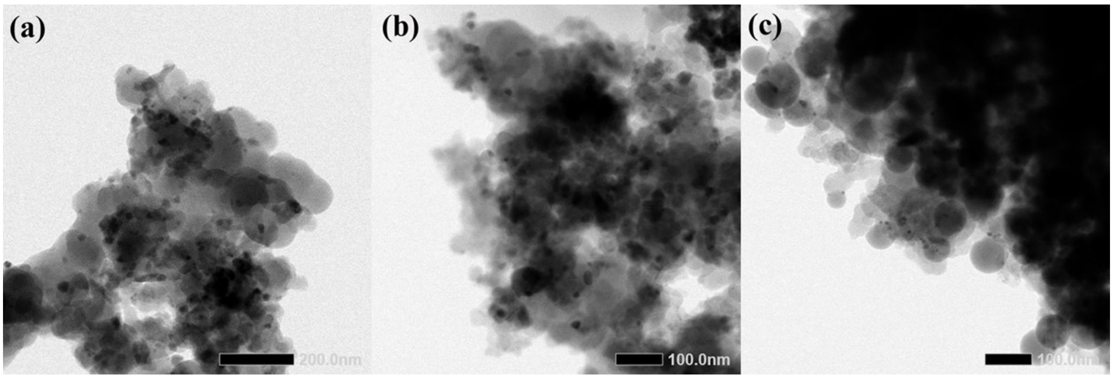

Fig. S3. TEM images of (a) RCB, (b) RCB600, (C)RCB1000.

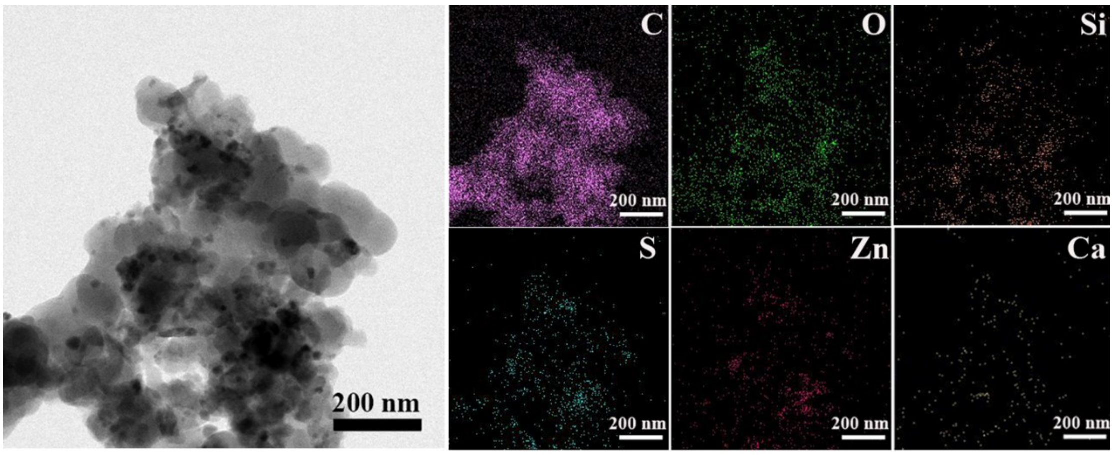

Fig. S4. TEM Elemental mapping images of RCB

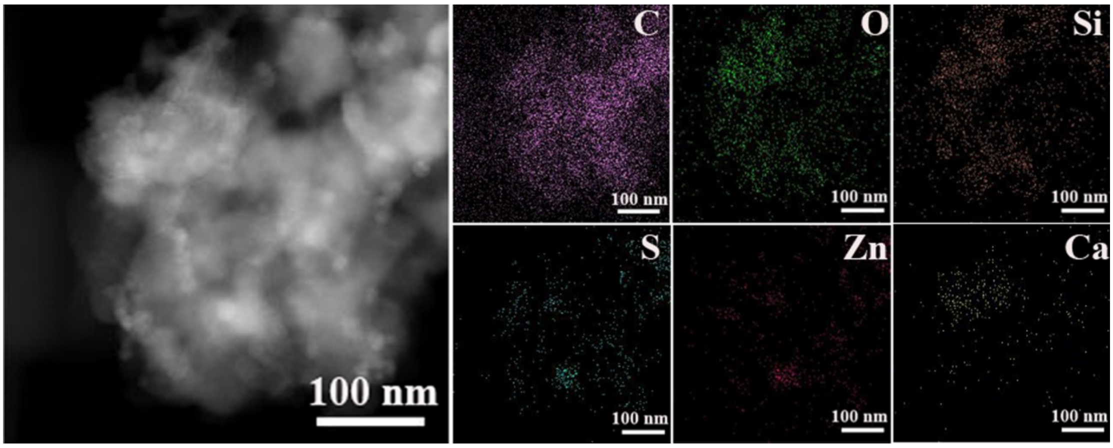

Fig. S5. TEM Elemental mapping images of RCB500.

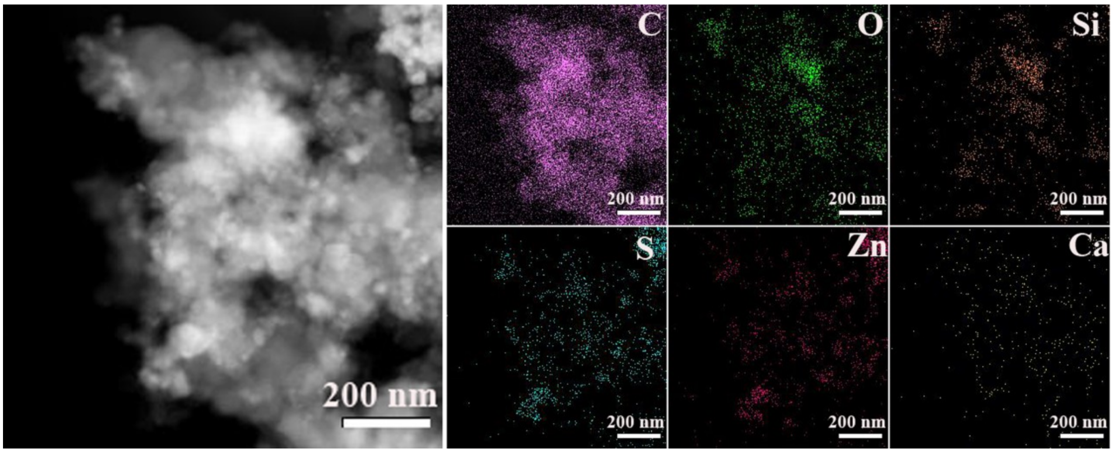

Fig. S6. TEM Elemental mapping images of RCB600.

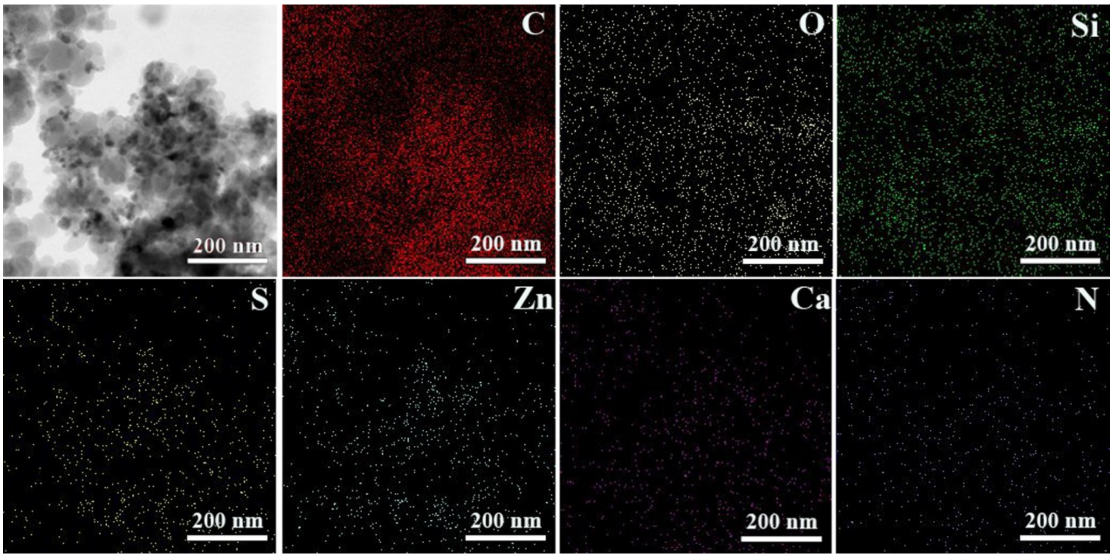

Fig. S7. TEM Elemental mapping images of RCB800.

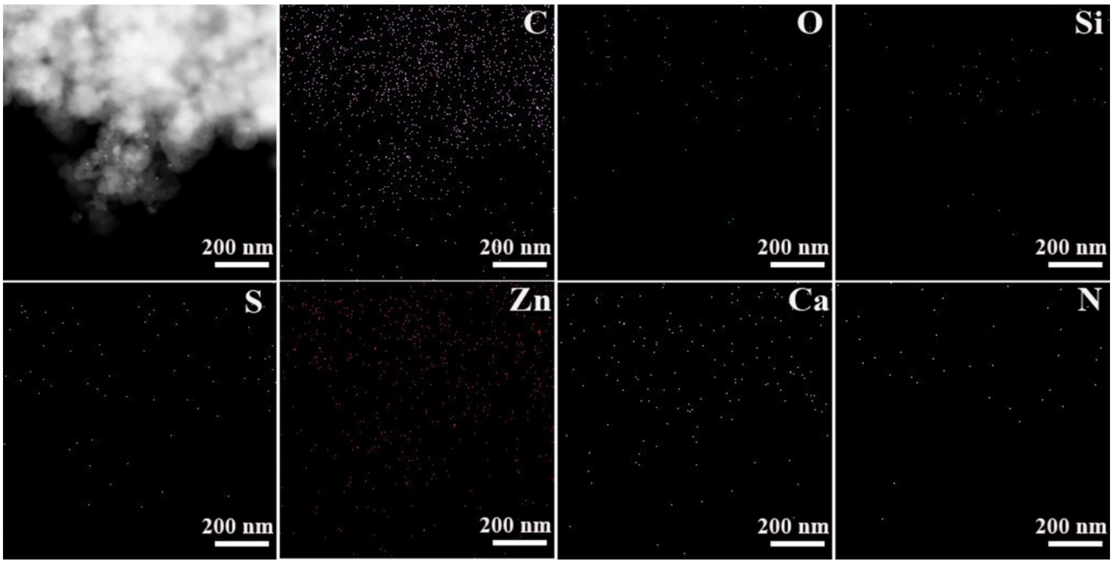

Fig. S8. TEM Elemental mapping images of RCB1000.

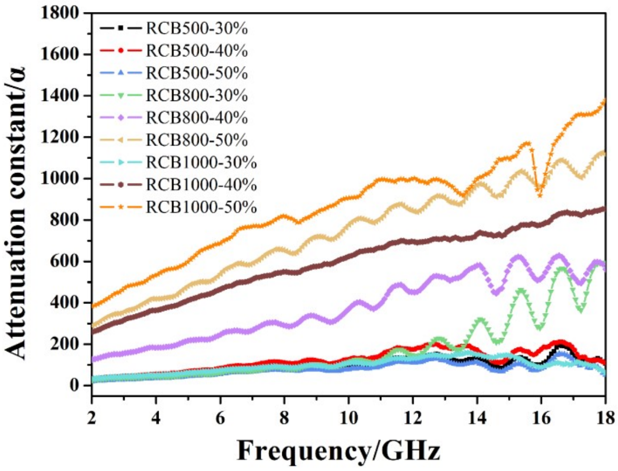

Fig. S9. Attenuation constants of all samples.
